# Multicomponent Synthesis and Evaluation of New 1,2,3-Triazole Derivatives of Dihydropyrimidinones as Acidic Corrosion Inhibitors for Steel

**DOI:** 10.3390/molecules21020250

**Published:** 2016-02-22

**Authors:** Rodrigo González-Olvera, Viridiana Román-Rodríguez, Guillermo E. Negrón-Silva, Araceli Espinoza-Vázquez, Francisco Javier Rodríguez-Gómez, Rosa Santillan

**Affiliations:** 1 Departamento de Ciencias Básicas, Universidad Autónoma Metropolitana-Azcapotzalco, Av. San Pablo No. 180, Ciudad de México, C.P. 02200, Mexico; rodrigo_glezo@hotmail.com (R.G.-O.); vacuidadd@gmail.com (V.R.-R.); 2 Departamento de Ingeniería Metalúrgica, Facultad de Química, Universidad Nacional Autónoma de México, C.U., Ciudad de México, C.P. 04510, Mexico; arasv_21@hotmail.com (A.E.-V.); fxavier@hotmail.com (F.J.R.-G.); 3 Departamento de Química, Centro de Investigación y de Estudios Avanzados del Instituto Politécnico Nacional, Apartado Postal 14-740, 07000 Ciudad de México, Mexico; rsantill@cinvestav.mx

**Keywords:** multicomponent synthesis, dihydropyrimidinone, 1,2,3-triazole, corrosion inhibitor, steel

## Abstract

An efficient one-pot synthesis of 1,2,3-triazole derivatives of dihydropyrimidinones has been developed using two multicomponent reactions. The aldehyde-1,2,3-triazoles were obtained in good yields from *in situ*-generated organic azides and *O*-propargylbenzaldehyde. The target heterocycles were synthesized through the Biginelli reaction in which the aldehyde-1,2,3-triazoles reacted with ethyl acetoacetate and urea in the presence of Ce(OTf)_3_ as the catalyst. The corrosion inhibition of steel grade API 5 L X52 in 1 M HCl by the synthesized compounds was investigated using the electrochemical impedance spectroscopy technique. The measurements revealed that these heterocycles are promising candidates to inhibit acidic corrosion of steel.

## 1. Introduction

**Scheme 1:**
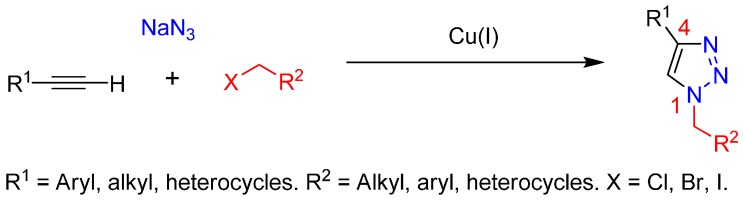
Multicomponent click reaction.

Multicomponent reactions (MCRs) are one-pot processes in which three or more starting materials are combined in a single reaction vessel to give a product that incorporates substantial portions of all the components. MCR processes are of great interest in organic and medicinal chemistry because of several attributes, including selectivity, atom economy, convergence, versatility, and molecular complexity [1,2,3,4,5,6]. In this regard, one example of MCR is the azide-alkyne 1,3-dipolar cycloaddition between benzyl halides, sodium azide and terminal alkyne to afforded 1,2,3-triazoles [7] (Scheme 1). Currently, the copper(I)-catalyzed azide-alkyne cycloaddition (also known as the Click or Huisgen-Meldal-Sharpless reaction) is the most widely used method for the synthesis of 1,4-disubstituted 1,2,3-triazoles from a wide range of organic azides and terminal alkynes [8,9,10].

**Figure 1:**
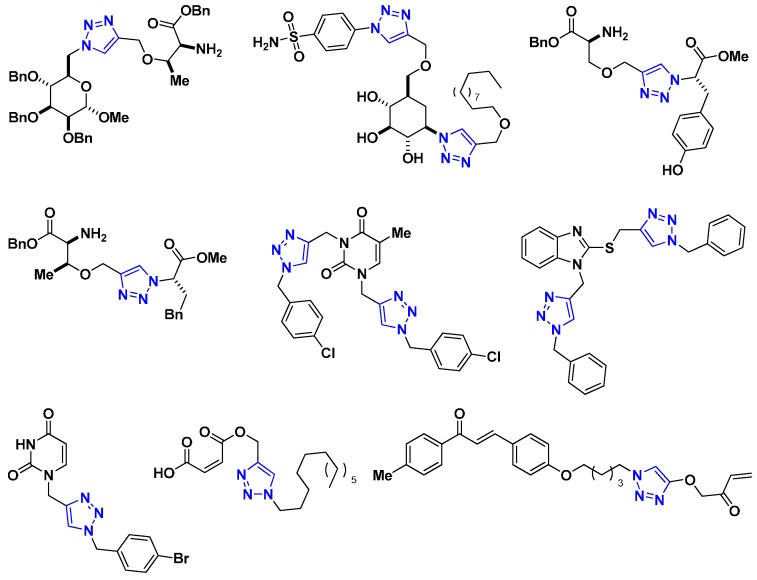
Representative 1,2,3-triazoles with corrosion inhibition activity.

Recently, 1,2,3-triazole derivatives of carbohydrates, amino acids, maleic acid, and chalcones have been studied as effective corrosion inhibitors of steel in acidic media (Figure 1) [11,12,13,14,15,16]. This nitrogen-containing heterocycle is not only used to mitigate corrosion but is also an important pharmacophore because of its presence in many compounds displaying pharmacological activities [17,18,19,20,21,22,23]. Additionally, our group has reported the multicomponent synthesis and corrosion inhibitory activity of 1,2,3-triazole derivatives of pyrimidine nucleobases and 2-mercaptobenzimidazole [24,25,26,27].

The corrosion of steels has received a considerable amount of attention as a result of industrial concern. API 5 L X52 steel is typically used in pipelines for gas and fuel conduction in the oil industry [28]. The steel pipelines for hydrocarbon transport are susceptible to corrosion cracking caused by hydrogen embrittlement and contact with fluids with high concentrations of chlorides [29,30]. One of the available methods to fight corrosion is the use of inhibitors that decrease the corrosion rates to an acceptable level. Corrosion inhibitors based on organic compounds containing nitrogen, oxygen, and sulfur atoms as well as π electrons associated with triple bonds, conjugated double bonds or aromatic rings are good inhibitors [31]. In this regard, 3,4-dihydropyrimidin-2(1*H*)-one (DHPM) is a heterocycle that possesses a wide range of biological activities [32,33,34,35,36,37]; however, its corrosion inhibition properties have hardly been studied [38].

Recently, several groups have introduced MCR strategies for the synthesis of heterocycles containing both dihydropyrimidinone and 1,2,3-triazole rings in their structures [39,40,41,42,43,44,45]. In continuation of our work on the development and study of organic corrosion inhibitors, we report herein a multicomponent synthesis of 1,2,3-triazole derivatives of dihydropyrimidinones and their evaluation as acidic corrosion inhibitors for API 5 L X52 steel.

## 2. Results and Discussion

### 2.1. Synthesis

**Scheme 2:**
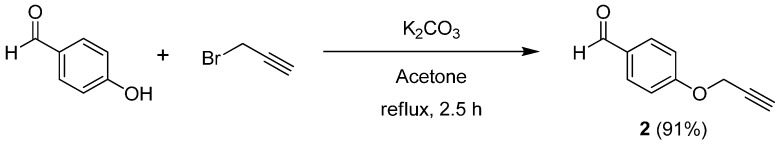
Synthesis of *O*-propargylbenzaldehyde (**2**).

For the synthesis of the title compounds, *O*-propargylbenzaldehyde (**2**) was first synthesized from 4-hydroxybenzaldehyde (**1**) and propargyl bromide in the presence of K_2_CO_3_ in acetone under reflux. After 2.5 h, the desired compound **2** was obtained in 91% yield after workup and purification by crystallization (Scheme 2).

Based on our previously reported methodology, we then performed the one-pot, three-component 1,3-dipolar cycloaddition reaction between *O*-propargylbenzaldehyde (**2**), sodium azide, and benzyl chloride in the presence of Cu(OAc)_2_·H_2_O, sodium ascorbate, and 1,10-phenantroline as the catalyst system in EtOH–H_2_O (2:1, *v*/*v*) at room temperature for 12 h [24,25,26]. The desired product **3** was obtained in 78% yield (Table 1, entry 1). As an alternative synthesis for this compound, we also performed the three-component reaction under microwave irradiation. When the reaction between the alkyne **2**, sodium azide, and benzyl chloride was performed under microwave irradiation, the aldehyde-triazole **3** was obtained in 82% yield after 15 min at 90 °C (Table 1, entry 2). The aldehyde-triazoles **4**–**7** were prepared under these established reaction conditions and were obtained in good yields (Table 1, entries 3–6).

The next step of our synthetic strategy for the production of 1,2,3-triazole-DHPMs **8**–**12** involved a Biginelli reaction. Many Lewis acids have been reported as efficient catalysts for the Biginelli reaction [36]. We performed the one-pot, three-component reaction between ethyl acetoacetate, the corresponding aldehyde-triazoles **3**–**7**, and urea using cerium trifluoromethanesulfonate [Ce(OTf)_3_] as a catalyst because of its high efficiency in this multicomponent reaction [46]. The heterocyclic compounds **8**–**12** were isolated in 80% to 87% yield (Table 2).

**Scheme 3:**
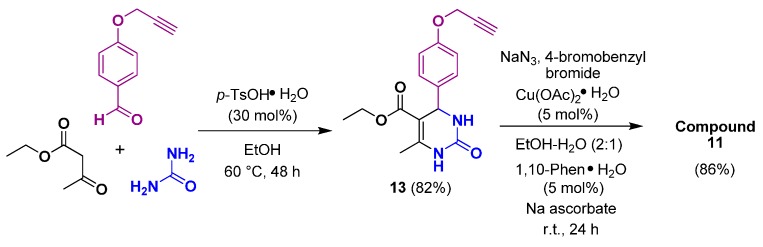
Synthesis of 1,2,3-triazole-DHPM through the Biginelli/Click sequence.

We also investigated another route for the synthesis of the target 1,2,3-triazole-DHPM, which involved the Biginelli/Click sequence. The preparation of DHPM (**13**) performed with ethyl acetoacetate, *O*-propargylbenzaldehyde (**2**), and urea in the presence of *p*-TsOH·H_2_O in EtOH at 60 °C for 48 h. The desired DHPM (**13**) was isolated in 82% yield and was used without purification for the following reaction. The ^1^H-NMR spectrum of DHPM (**13**) exhibited a triplet at δ = 3.52 (≡CH) and a doublet at δ = 4.76 (CH_2_) for the propargyl fragment, whereas signals at δ = 55.9 (CH_2_), 78.5 (≡CH), and 79.8 (C≡) appeared in the ^13^C-NMR spectrum (Figure S11). The one-pot, three-component reaction between the alkyne (**13**), 4-bromobenzyl bromide, and sodium azide was performed in the presence of a copper catalyst to give DHPM-triazole (**11**) in 86% yield (Scheme 3).

It is worth noting that the Biginelli reaction and the Click reaction cannot be performed directly in one step. When all of the reactants—*O*-propargylbenzaldehyde (**2**), 4-bromobenzyl bromide, sodium azide, ethyl acetoacetate and urea—were stirred in EOH–H_2_O (1:1 *v*/*v*) at 90 °C for 48 h in the presence of Cu(OAc)_2_·H_2_O (10 mol %), sodium ascorbate, and 1,10-phenantroline, only the aldehyde-triazole **6** was observed by thin layer chromatography (CH_2_Cl_2_–EtOH 95:5 *v*/*v*).

The structures of the synthesized heterocycles **8**–**12** were confirmed by examination of their ^1^H- and ^13^C-NMR and high-resolution mass spectra. The ^1^H- and ^13^C-NMR signals for compounds **8**–**12** were assigned with the help of 2D heteronuclear correlation experiments (HSQC and HMBC). The signals in the ^1^H-NMR spectra at δ = 8.27–8.28 corresponded to the triazolyl hydrogen, which were supported by the signals in the ^13^C-NMR spectra at δ = 125.0–125.1. The signals for the quaternary carbon of the triazole ring appeared at δ = 143.6–143.6 in the ^13^C-NMR spectra. These chemical shift values are consistent with those reported for 1,4-disubstituted 1,2,3-triazoles [24,25,26].

### 2.2. Corrosion Inhibition Efficiencies

**Figure 2:**
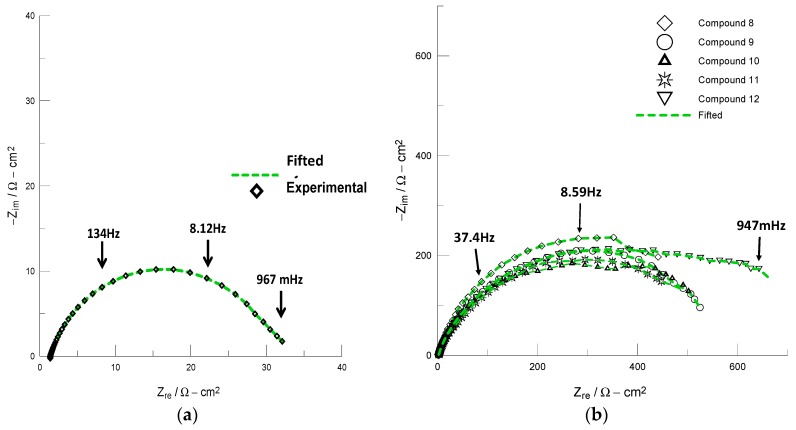
Nyquist plots recorded in the following systems: (**a**) API 5 L X52/1 M HCl and (**b**) API 5 L X52/1 M HCl +10 ppm of compounds **8**–**12**.

We employed electrochemical impedance spectroscopy (EIS) to evaluate the activity of compounds (**8**–**12**) on the corrosion inhibition of API 5 L X52 steel. The Nyquist plots obtained for API 5 L X52 steel in 1 M HCl solution in the absence and presence of the tested compounds **8**–**12** are shown in Figure 2. Figure 2a clearly shows that the steel in 1 M HCl shows one semicircle (Z_re_~30 Ωcm^2^), which indicates that the steel corrosion is mainly controlled by a charge transfer process. In contrast, the corresponding Nyquist plots obtained for the steel in the acid solution in the presence of **8**–**12** (10 ppm) increased the impedance (Z_re_) value (Figure 2b), which is generally attributed to the adsorption of the organic compounds onto the metal surface [16,27]. The electrochemical parameters obtained from fitting the recorded EIS data using the appropriate equivalent circuit model (R(Q)R) are listed in Table 3.

The electrochemical data in Table 3 show that the charge transfer resistance (R_ct_) values increased, whereas the double layer capacitance (C_dl_) values decreased with addition of compounds **8**–**12**. A large R_ct_ value is associated with a slower corrosion rate, whereas the decrease in C_dl_ can be attributed to the formation of a protective layer on the metal surface [14,16,27]. It is worth noting that all of the compounds exhibited corrosion inhibitory activity with inhibition efficiencies (IE) of approximately 95% at relatively low concentration values. These compounds provide excellent inhibition activity under static conditions that is comparable to if not better than those of organic inhibitors that incorporate the 1,2,3-triazole moiety [11,12,13,14,15,16,25,26,27].

## 3. Experimental Section

### 3.1. General Information

Commercially available reagents and solvents were used as received. Column chromatography was performed on Kieselgel silica gel 60 (230–400 mesh). Melting points were determined using a Fisher-Johns apparatus and are uncorrected. IR spectra were recorded on an Alpha FT-IR/ATR spectrometer (Bruker, Leipzig, Germany). The NMR spectra (400 MHz for ^1^H and 100.6 MHz for ^13^C) were obtained using a Bruker Ascend-400 spectrometer (Billerica, MA, USA). Chemical shifts (δ) are given in ppm and coupling constants (*J*) are given in hertz (Hz). High-resolution mass spectra (HRMS) were recorded on JMS-SX 102a (JEOL, Tokyo, Japan) and MSD-TOF-1069A (Agilent, Santa Clara, CA, USA) spectrometers. Microwave irradiation experiments were performed using a Discover System (CEM Corporation, Matthews, NC, USA) single-mode microwave with standard sealed microwave glass vials. The reaction temperature was monitored by an IR sensor on the outside wall of the reaction vial. The electrochemical impedance study was performed at room temperature using a Gill-AC electrochemical workstation (ACM Instruments, Cark, Cumbria, UK), with a sinusoidal perturbation signal of ±10 mV around E_corr_, within a range of 10^−1^ Hz to 10^4^ Hz. A saturated Ag/AgCl electrode was used as the reference, a graphite rod was used as the counter electrode, and the working electrode was the API 5 L X52 steel sample with an exposed area of approximately 1 cm^2^ and a chemical composition (wt %) of C: 0.080, Mn: 1.06, Si: 0.26, Ti: 0.003, V: 0.054, Nb: 0.041, P: 0.019, S: 0.003, Al: 0.039, Ni: 0.019, C_eq_: 0.274, Fe: balance [47], which was prepared prior to each experiment using standard metallographic procedures. The corrosion inhibition efficiency (IE) was evaluated by means of electrochemical impedance spectroscopy (EIS) in the API 5 L X52 steel/1 M HCl system containing 0 (blank) or 10 ppm of the organic inhibitor. A simulation of the impedance data recorded was conducted by means of electrical equivalent circuits and the electrical parameters: charge transfer resistance (R_ct_) and double layer capacitance (C_dl_) were obtained in this way.

### 3.2. Product Synthesis and Characterization

*4-(Prop-2-ynyloxy)benzaldehyde* (**2**). In a 100 mL round bottom flask equipped with a magnetic stirrer and a reflux condenser, 4-hydroxybenzaldehyde (**1**, 1.00 g, 8.18 mmol) was dissolved in acetone (20 mL). Anhydrous potassium carbonate (1.47 g, 10.63 mmol) was then added and the mixture was heated to reflux for 30 min. After cooling to room temperature, propargyl bromide (1.18 mL, 13.29 mmol, 80 wt.% in toluene) was added slowly and the reaction mixture was heated to reflux for an additional 2 h. The solvent was evaporated under vacuum and the crude reaction mixture was treated with H_2_O (15 mL) and extracted with EtOAc (3 × 15 mL). The combined organic phases were dried (Na_2_SO_4_) and then activated charcoal was added and the combined organics were stirred and heated at 40 °C for 15 min. After the combined organics were filtered over Celite, which was washed with EtOAc (15 mL), the solvent was evaporated under vacuum and the crude product was purified by recrystallization from EtOAc/hexane (1:2 *v*/*v*) to afford 1.19 g (91% yield) of **2** as a white solid, m.p. 75–77 °C (Lit. [48] m.p. 75–78 °C). ^1^H-NMR (CDCl_3_): δ = 2.59 (t, *J* = 2.4 Hz, 1H, ≡CH), 4.79 (d, *J* = 2.4 Hz, 2H, OCH_2_), 7.10 (d, *J* = 8.7 Hz, 2H, ArH), 7.87 (d, *J* = 8.8 Hz, 2H, ArH), 9.91 (s, 1H, CHO). ^13^C-NMR (CDCl_3_): δ = 55.9 (OCH_2_), 76.3 (≡CH), 77.5 (C≡), 115.2 (2 × ArCH), 130.6 (C_ipso_), 131.9 (2 × ArCH), 162.4 (O-C_ipso_), 190.7 (C=O). FT-IR/ATR ν_max_ cm^−1^: 3206 (≡CH), 2831, 2807, 2749, 2121 (C≡C), 1677 (C=O), 1601, 1574, 1504, 1248, 1213, 1168, 1006, 825, 509. HRMS (ESI-TOF) calculated for C_10_H_8_O_2_ + H^+^: 161.0597; Found: 161.0599.

*4-((1-Benzyl-1H-1,2,3-triazol-4-yl)methoxy)benzaldehyde* (**3**). In a 10 mL microwave vial equipped with a magnetic stirrer, Cu(OAc)_2_·H_2_O (11.4 mg, 0.063 mmol, 5 mol%), 1,10-phenanthroline monohydrate (12.5 mg, 0.063 mmol, 5 mol%), and sodium l-ascorbate (188 mg, 0.95 mmol) were added in EtOH–H_2_O (1:1 *v*/*v*, 4 mL), followed by stirring for five min at room temperature. Subsequently, alkyne **2** (200 mg, 1.25 mmol), sodium azide (90 mg, 1.38 mmol), and benzyl chloride (0.16 mL, 1.38 mmol) were added to the reaction mixture, which was irradiated (5–6 W) at 90 °C for 15 min. Afterward, H_2_O (15 mL) was added to the reaction mixture and extracted with CH_2_Cl_2_ (3 × 15 mL). The combined organic phases were dried (Na_2_SO_4_) and then the solvent was evaporated under vacuum. The crude product was purified by column chromatography (Hexane/EtOAc 1:1, *v*/*v*) and recrystallized from EtOAc/hexane (1:2, *v*/*v*) to afford 300 mg (82% yield) of **3** as a white solid, m.p. 95–97 °C [Lit. [48] m.p.100–103 °C]. ^1^H-NMR (CDCl_3_): δ = 5.26 (s, 2H, OCH_2_), 5.54 (s, 2H, NCH_2_), 7.08 (d, *J* = 8.7 Hz, 2H, ArH), 7.26–7.29 (m, 2H, ArH), 7.36–7.39 (m, 3H, ArH), 7.55 (s, 1H, ArH triazole), 7.82 (d, *J* = 8.7 Hz, 2H, ArH), 9.88 (s, 1H, CHO). ^13^C-NMR (CDCl_3_): δ = 54.3 (NCH_2_), 62.2 (OCH_2_), 115.0 (2 × ArCH), 122.8 (ArCH triazole), 128.2 (2 × ArCH), 128.9 (ArCH), 129.2 (2 × ArCH), 130.4 (C_ipso_), 132.0 (2 × ArCH), 134.3 (C_ipso_), 143.6 (C_ipso_ triazole), 163.1 (O-C_ipso_), 190.8 (C=O). FT-IR/ATR ν_max_ cm^−1^: 3143, 3097, 3064, 2963, 2935, 2842, 2820, 2760, 1673 (C=O), 1601, 1575, 1240, 1216, 1172, 834, 752. HRMS (ESI-TOF) calculated for C_17_H_15_N_3_O_2_ + H^+^: 294.1237; Found: 294.1240.

*4-((1-(4-Fluorobenzyl)-1H-1,2,3-triazol-4-yl)methoxy)benzaldehyde* (**4**). The procedure described above (using the same quantities of Cu(OAc)_2_·H_2_O, 1,10-phenanthroline monohydrate, and sodium L-ascorbate) was followed to obtain compound **4**, employing alkyne **2** (200 mg, 1.25 mmol), NaN_3_ (90 mg, 1.38 mmol), and 4-fluorobenzyl chloride (0.16 mL, 1.38 mmol). The crude product was purified by column chromatography (hexane/EtOAc 1:1 *v*/*v*) and recrystallized from EtOAc/hexane (1:2 *v*/*v*) to afford 313 mg (80% yield) of the desired product **4** as a white solid, m.p. 89–90 °C [Lit. [49] m.p.101–104 °C]. ^1^H-NMR (CDCl_3_): δ = 5.26 (s, 2H, OCH_2_), 5.51 (s, 2H, NCH_2_), 7.04–7.09 (m, 4H, ArH), 7.26–7.30 (m, 2H, ArH), 7.56 (s, 1H, ArH triazole), 7.83 (d, *J* = 8.8 Hz, 2H, ArH), 9.88 (s, 1H, CHO). ^13^C-NMR (CDCl_3_): δ = 53.6 (NCH_2_), 62.2 (OCH_2_), 115.1 (2 × ArCH), 116.2 (d, *J*_CF_ = 21.8 Hz, 2 × ArCH), 122.6 (ArCH triazole), 130.1 (d, *J*_CF_ = 8.4 Hz, 2 × ArCH), 130.2 (d, *J*_CF_ = 3.3 Hz, C_ipso_), 130.4 (C_ipso_), 132.0 (2 × ArCH), 143.8 (C_ipso_ triazole), 162.9 (d, *J*_CF_ = 248.5 Hz, F-C_ipso_), 163.1 (O-C_ipso_), 190.7 (C=O). FT-IR/ATR ν_max_ cm^−1^: 3151, 3068, 3038, 2944, 2841, 2759, 1672 (C=O), 1601, 1574, 1456, 1254, 1217, 1157, 1032, 823, 501. HRMS (ESI-TOF) calculated for C_17_H_14_FN_3_O_2_ +H^+^: 312.1143; Found: 312.1146.

*4-((1-(4-Chlorobenzyl)-1H-1,2,3-triazol-4-yl)methoxy)benzaldehyde* (**5**). The procedure described above (using the same quantities of Cu(OAc)_2_·H_2_O, 1,10-phenanthroline monohydrate, and sodium l-ascorbate) was followed to obtain compound **5**, employing alkyne **2** (200 mg, 1.25 mmol), NaN_3_ (90 mg, 1.38 mmol), and 4-chlorobenzyl chloride (222 mg, 1.38 mmol). The crude product was purified by column chromatography (hexane/EtOAc 1:1, *v*/*v*) and recrystallized from EtOAc/hexane (1:2, *v*/*v*) to afford 350 mg (85% yield) of the desired product **5** as a white solid, m.p. 88–89 °C [Lit. [49] m.p.108–110 °C]. ^1^H-NMR (CDCl_3_): δ = 5.26 (s, 2H, OCH_2_), 5.52 (s, 2H, NCH_2_), 7.08 (d, *J* = 8.7 Hz, 2H, ArH), 7.22 (d, *J* = 8.4 Hz, 2H, ArH), 7.35 (d, *J* = 8.4 Hz, 2H, ArH), 7.58 (s, 1H, ArH triazole), 7.83 (d, *J* = 8.7 Hz, 2H, ArH), 9.88 (s, 1H, CHO). ^13^C-NMR (CDCl_3_): δ = 53.6 (NCH_2_), 62.1 (OCH_2_), 115.1 (2 × ArCH), 122.8 (ArCH triazole), 129.4 (2 × ArCH), 129.5 (2 × ArCH), 130.4 (C_ipso_), 132.0 (2 × ArCH), 132.8 (Cl-C_ipso_), 135.0 (C_ipso_), 143.8 (C_ipso_ triazole), 163.1 (O-C_ipso_), 190.8 (C=O). FT-IR/ATR ν_max_ cm^−1^: 3149, 3100, 3055, 2943, 2807, 2744, 1671 (C=O), 1600, 1571, 1160, 992, 833, 806, 772, 501. HRMS (ESI-TOF) calculated for C_17_H_14_ClN_3_O_2_ + H^+^: 328.0847; Found: 328.0845.

*4-((1-(4-Bromobenzyl)-1H-1,2,3-triazol-4-yl)methoxy)benzaldehyde* (**6**). The procedure described above (using the same quantities of Cu(OAc)_2_·H_2_O, 1,10-phenanthroline monohydrate, and sodium l-ascorbate) was followed to obtain compound **6**, employing alkyne **2** (200 mg, 1.25 mmol), NaN_3_ (90 mg, 1.38 mmol), and 4-bromobenzyl bromide (345 mg, 1.38 mmol). The crude product was purified by column chromatography (hexane/EtOAc 1:1, *v*/*v*) and recrystallized from EtOAc/hexane (1:2, *v*/*v*) to afford 355 mg (76% yield) of the desired product **6** as a white solid, m.p. 82–84 °C. ^1^H-NMR (CDCl_3_): δ = 5.26 (s, 2H, OCH_2_), 5.50 (s, 2H, NCH_2_), 7.08 (d, *J* = 8.8 Hz, 2H, ArH), 7.16 (d, *J* = 8.4 Hz, 2H, ArH), 7.51 (d, *J* = 8.4 Hz, 2H, ArH), 7.57 (s, 1H, ArH triazole), 7.83 (d, *J* = 8.8 Hz, 2H, ArH), 9.88 (s, 1H, CHO). ^13^C-NMR (CDCl_3_): δ = 53.6 (NCH_2_), 62.2 (OCH_2_), 115.1 (2 × ArCH), 122.7 (ArCH triazole), 123.1 (Br-C_ipso_), 129.7 (2 × ArCH), 130.4 (C_ipso_), 132.0 (2 × ArCH), 132.4 (2 × ArCH), 133.3 (C_ipso_), 143.9 (C_ipso_ triazole), 163.1 (O-C_ipso_), 190.7 (C=O). FT-IR/ATR ν_max_ cm^−1^: 3149, 3100, 3054, 2943, 2807, 2746, 1670 (C=O), 1600, 1572, 1249, 1212, 1160, 991, 833, 770, 491. HRMS (ESI-TOF) calculated for C_17_H_14_BrN_3_O_2_ + H^+^: 372.0342; Found: 372.0347.

*4-((1-(4-Iodobenzyl)-1H-1,2,3-triazol-4-yl)methoxy)benzaldehyde* (**7**). The procedure described above was followed to obtain compound **7**, employing Cu(OAc)_2_·H_2_O (5.6 mg, 0.031 mmol), 1,10-phenanthroline monohydrate (6.1 mg, 0.031 mmol), sodium L-ascorbate (93 mg, 0.47 mmol), alkyne **2** (100 mg, 0.62 mmol), NaN_3_ (44 mg, 0.68 mmol), and 4-iodobenzyl bromide (214 mg, 0.72 mmol). The crude product was purified by column chromatography (Hexane/EtOAc 1:1 *v*/*v*) and recrystallized from EtOAc/hexane (1:2 *v*/*v*) to afford 190 mg (73% yield) of the desired product **7** as a white solid, m.p. 85–87 °C. ^1^H-NMR (CDCl_3_): δ = 5.27 (s, 2H, OCH_2_), 5.49 (s, 2H, NCH_2_), 7.03 (d, *J* = 8.4 Hz, 2H, ArH), 7.08 (d, *J* = 8.7 Hz, 2H, ArH), 7.56 (s, 1H, ArH triazole), 7.71 (d, *J* = 8.4 Hz, 2H, ArH), 7.83 (d, *J* = 8.8 Hz, 2H, ArH), 9.88 (s, 1H, CHO). ^13^C-NMR (CDCl_3_): δ = 53.7 (NCH_2_), 62.2 (OCH_2_), 94.7 (I-C_ipso_), 115.1 (2 × ArCH), 122.7 (ArCH triazole), 129.9 (2 × ArCH), 130.4 (C_ipso_), 132.0 (2 × ArCH), 134.0 (C_ipso_), 138.4 (2 × ArCH), 143.9 (C_ipso_ triazole), 163.1 (O-C_ipso_), 190.7 (C=O). FT-IR/ATR ν_max_ cm^−1^: 3154, 2953, 2919, 2873, 2820, 2799, 2738, 1682 (C=O), 1604, 1579, 1252, 1210, 1154, 1047, 1008, 811, 768. HRMS (ESI-TOF) calculated for C_17_H_14_IN_3_O_2_ + H^+^: 420.0203; Found: 420.0206.

*Ethyl 4-(4-((1-benzyl-1H-1,2,3-triazol-4-yl)methoxy)phenyl)-6-methyl-2-oxo-1,2,3,4-tetrahydropyrimidine-5-carboxylate* (**8**). In a 25 mL round-bottomed flask equipped with a magnetic stirrer, aldehyde-triazole **3** (100 mg, 0.34 mmol), urea (22 mg, 0.37 mmol), ethyl acetoacetate (47 μL, 0.37 mmol), and cerium trifluoromethanesulfonate (40 mg, 0.068 mmol, 20 mol %) were added in EtOH (2 mL). The reaction mixture was stirred at room temperature for 3 days. The solvent was evaporated under vacuum and the crude reaction mixture was purified by column chromatography (CH_2_Cl_2_→CH_2_Cl_2_/EtOH 98:2 *v*/*v*) to afford 122 mg (80% yield) of **8** as a white foam, m.p. 72–75 °C [Lit. [41] m.p.131–133 °C]. ^1^H-NMR (DMSO-*d*_6_): δ = 1.09 (t, *J* = 7.1 Hz, 3H, CH_3_), 2.24 (s, 3H, CH_3_), 3.97 (q, *J* = 7.1 Hz, 2H, OCH_2_), 5.09 (s, 1H, CH), 5.10 (s, 2H, OCH_2_), 5.61 (s, 2H, NCH_2_), 6.97 (d, *J* = 8.7 Hz, 2H, ArH), 7.15 (d, *J* = 8.7 Hz, 2H, ArH), 7.30–7.40 (m, 5H, ArH), 7.70 (br, 1H, NH), 8.28 (s, 1H, ArH triazole), 9.18 (br, 1H, NH). ^13^C-NMR (DMSO-*d*_6_): δ = 14.6 (OCH_2_*C*H_3_), 18.2 (CH_3_), 53.3 (NCH_2_), 53.8 (CH), 59.6 (O*C*H_2_CH_3_), 61.5 (OCH_2_), 100.0 (C=), 114.9 (2 × ArCH), 125.1 (ArCH triazole), 127.9 (2 × ArCH), 128.4 (2 × ArCH), 128.6 (ArCH), 129.2 (2 × ArCH), 136.5 (C_ipso_), 137.9 (C_ipso_), 143.5 (C_ipso_ triazole), 148.5 (=CNH), 152.6 (NC=ON), 157.7 (O-C_ipso_), 165.8 (OC=O). FT-IR/ATR ν_max_ cm^−1^: 3231, 3098, 2931, 1692 (C=O), 1638, 1507, 1454, 1220, 1174, 1085, 794, 756, 716, 653. HRMS (ESI-TOF) calculated for C_24_H_25_N_5_O_4_ + H^+^: 448.1979; Found: 448.1983.

*Ethyl 4-(4-((1-(4-fluorobenzyl)-1H-1,2,3-triazol-4-yl)methoxy)phenyl)-6-methyl-2-oxo-1,2,3,4-tetrahydro-pyrimidine-5-carboxylate* (**9**). The procedure described above was followed to obtain compound **9**, employing aldehyde-triazole **4** (100 mg, 0.32 mmol), urea (21 mg, 0.35 mmol), ethyl acetoacetate (44 μL, 0.35 mmol), and cerium trifluoromethanesulfonate (38 mg, 0.064 mmol, 20 mol %). The crude reaction mixture was purified by column chromatography (CH_2_Cl_2_→CH_2_Cl_2_/EtOH 98:2 *v*/*v*) to afford 130 mg (87% yield) of **9** as a white foam, m.p. 77–80 °C. ^1^H-NMR (DMSO-*d*_6_): δ = 1.10 (t, *J* = 7.1 Hz, 3H, CH_3_), 2.25 (s, 3H, CH_3_), 3.98 (q, *J* = 7.1 Hz, 2H, OCH_2_), 5.10 (s, 3H, CH, OCH_2_), 5.60 (s, 2H, NCH_2_), 6.97 (d, *J* = 8.6 Hz, 2H, ArH), 7.16 (d, *J* = 8.1 Hz, 2H, ArH), 7.18–7.23 (m, 2H, ArH), 7.38–7.42 (m, 2H, ArH), 7.67 (br, 1H, NH), 8.27 (s, 1H, ArH triazole), 9.15 (br, 1H, NH). ^13^C-NMR (DMSO-*d*_6_): δ = 14.5 (OCH_2_*C*H_3_), 18.2 (CH_3_), 52.5 (NCH_2_), 53.8 (CH), 59.6 (O*C*H_2_CH_3_), 61.6 (OCH_2_), 100.1 (C=), 115.0 (2 × ArCH), 116.0 (d, *J*_CF_ = 21.6 Hz, 2 × ArCH), 125.0 (ArCH triazole), 127.9 (2 × ArCH), 130.7 (d, *J*_CF_ = 8.4 Hz, 2 × ArCH), 132.6 (d, *J*_CF_ = 3.0 Hz, C_ipso_), 137.9 (C_ipso_), 143.6 (C_ipso_ triazole), 148.5 (=CNH), 152.6 (NC=ON), 157.7 (O-C_ipso_), 162.4 (d, *J*_CF_ = 244.5 Hz, F-C_ipso_), 165.9 (OC=O). FT-IR/ATR ν_max_ cm^−1^: 3228, 3098, 2928, 1692 (C=O), 1639, 1508, 1455, 1220, 1085, 829, 778. HRMS (ESI-TOF) calculated for C_24_H_24_FN_5_O_4_ + H^+^: 466.1885; Found: 466.1889.

*Ethyl 4-(4-((1-(4-chlorobenzyl)-1H-1,2,3-triazol-4-yl)methoxy)phenyl)-6-methyl-2oxo-1,2,3,4-tetrahydro-pyrimidine-5-carboxylate* (**10**). The procedure described above was followed to obtain compound **10**, employing aldehyde-triazole **5** (100 mg, 0.30 mmol), urea (20 mg, 0.33 mmol), ethyl acetoacetate (42 μL, 0.33 mmol), and cerium trifluoromethanesulfonate (35 mg, 0.060 mmol, 20 mol %). The crude reaction mixture was purified by column chromatography (CH_2_Cl_2_→CH_2_Cl_2_/EtOH 98:2, *v*/*v*) to afford 125 mg (86% yield) of **10** as a white solid, m.p. 157–158 °C. ^1^H-NMR (DMSO-*d*_6_): δ = 1.09 (t, *J* = 7.1 Hz, 3H, CH_3_), 2.24 (s, 3H, CH_3_), 3.97 (q, *J* = 7.1 Hz, 2H, OCH_2_), 5.09 (s, 1H, CH), 5.10 (s, 2H, OCH_2_), 5.61 (s, 2H, NCH_2_), 6.97 (d, *J* = 8.7 Hz, 2H, ArH), 7.15 (d, *J* = 8.6 Hz, 2H, ArH), 7.34 (d, *J* = 8.5 Hz, 2H, ArH), 7.44 (d, *J* = 8.4 Hz, 2H, ArH), 7.67 (br, 1H, NH), 8.28 (s, 1H, ArH triazole), 9.15 (br, 1H, NH). ^13^C-NMR (DMSO-*d*_6_): δ = 14.6 (OCH_2_*C*H_3_), 18.2 (CH_3_), 52.5 (NCH_2_), 53.8 (CH), 59.6 (O*C*H_2_CH_3_), 61.5 (OCH_2_), 100.0 (C=), 114.9 (2 × ArCH), 125.1 (ArCH triazole), 127.9 (2 × ArCH), 129.2 (2 × ArCH), 130.4 (2 × ArCH), 133.4 (Cl-C_ipso_), 135.5 (C_ipso_), 137.9 (C_ipso_), 143.5 (C_ipso_ triazole), 148.5 (=CNH), 152.6 (NC=ON), 157.7 (O-C_ipso_), 165.8 (OC=O). FT-IR/ATR ν_max_ cm^−1^: 3197, 3085, 2926, 1697 (C=O), 1638, 1218, 1172, 1090, 1017, 767. HRMS (ESI-TOF) calculated for C_24_H_24_ClN_5_O_4_ + H^+^: 482.1589; Found: 482.1595.

*Ethyl 4-(4-((1-(4-bromobenzyl)-1H-1,2,3-triazol-4-yl)methoxy)phenyl)-6-methyl-2-oxo-1,2,3,4-tetrahydro-pyrimidine-5-carboxylate* (**11**). The procedure described above was followed to obtain compound **11**, employing aldehyde-triazole **6** (100 mg, 0.27 mmol), urea (18 mg, 0.30 mmol), ethyl acetoacetate (38 μL, 0.30 mmol), and cerium trifluoromethanesulfonate (32 mg, 0.054 mmol, 20 mol %). The crude reaction mixture was purified by column chromatography (CH_2_Cl_2_→CH_2_Cl_2_/EtOH 98:2, *v*/*v*) to afford 120 mg (85% yield) of **11** as a white solid, m.p. 188–190 °C. ^1^H-NMR (DMSO-*d*_6_): δ = 1.10 (t, *J* = 7.1 Hz, 3H, CH_3_), 2.25 (s, 3H, CH_3_), 3.98 (q, *J* = 7.1 Hz, 2H, OCH_2_), 5.10 (s, 3H, CH, OCH_2_), 5.60 (s, 2H, NCH_2_), 6.97 (d, *J* = 8.6 Hz, 2H, ArH), 7.15 (d, *J* = 8.6 Hz, 2H, ArH), 7.28 (d, *J* = 8.3 Hz, 2H, ArH), 7.58 (d, *J* = 8.4 Hz, 2H, ArH), 7.66 (br, 1H, NH), 8.28 (s, 1H, ArH triazole), 9.14 (br, 1H, NH). ^13^C-NMR (DMSO-*d*_6_): δ = 14.6 (OCH_2_*C*H_3_), 18.2 (CH_3_), 52.6 (NCH_2_), 53.8 (CH), 59.6 (O*C*H_2_CH_3_), 61.6 (OCH_2_), 100.0 (C=), 115.0 (2 × ArCH), 121.9 (Br-C_ipso_), 125.1 (ArCH triazole), 127.9 (2 × ArCH), 130.7 (2 × ArCH), 132.2 (2 × ArCH), 135.8 (C_ipso_), 137.9 (C_ipso_), 143.6 (C_ipso_ triazole), 148.5 (=CNH), 152.6 (NC=ON), 157.7 (O-C_ipso_), 165.8 (OC=O). FT-IR/ATR ν_max_ cm^−1^: 3197, 3086, 2925, 1696 (C=O), 1638, 1218, 1089, 826, 766, 656. HRMS (ESI-TOF) calculated for C_24_H_24_BrN_5_O_4_ + H^+^: 526.1084; Found: 526.1086.

*Ethyl 4-(4-((1-iodobenzyl)-1H-1,2,3-triazol-4-yl)methoxy)phenyl)-6-methyl-2-oxo-1,2,3,4-tetrahydro-pyrimidine-5-carboxylate* (**12**). The procedure described above was followed to obtain compound **12**, employing aldehyde-triazole **7** (100 mg, 0.24 mmol), urea (16 mg, 0.26 mmol), ethyl acetoacetate (33 μL, 0.26 mmol), and cerium trifluoromethanesulfonate (28 mg, 0.048 mmol, 20 mol %). The crude reaction mixture was purified by column chromatography (CH_2_Cl_2_→CH_2_Cl_2_/EtOH 98:2, *v*/*v*) to afford 115 mg (83% yield) of **12** as a white solid, m.p. 208–210 °C. ^1^H-NMR (DMSO-*d*_6_): δ = 1.10 (t, *J* = 7.1 Hz, 3H, CH_3_), 2.24 (s, 3H, CH_3_), 3.97 (q, *J* = 7.1 Hz, 2H, OCH_2_), 5.10 (s, 3H, CH, OCH_2_), 5.57 (s, 2H, NCH_2_), 6.97 (d, *J* = 8.7 Hz, 2H, ArH), 7.11–7.16 (m, 4H, ArH), 7.70 (br, 1H, NH), 7.75 (d, *J* = 8.2 Hz, 2H, ArH), 8.28 (s, 1H, ArH triazole), 9.18 (br, 1H, NH). ^13^C-NMR (DMSO-*d*_6_): δ = 14.6 (OCH_2_*C*H_3_), 18.2 (CH_3_), 52.7 (NCH_2_), 53.8 (CH), 59.6 (O*C*H_2_CH_3_), 61.5 (OCH_2_), 94.9 (I-C_ipso_), 100.0 (C=), 114.9 (2 × ArCH), 125.1 (ArCH triazole), 127.9 (2 × ArCH), 130.7 (2 × ArCH), 136.2 (C_ipso_), 137.9 (C_ipso_), 138.0 (2 × ArCH), 143.5 (C_ipso_ triazole), 148.5 (=CNH), 152.6 (NC=ON), 157.7 (O-C_ipso_), 165.8 (OC=O). FT-IR/ATR ν_max_ cm^−1^: 3212, 3085, 2929, 1692 (C=O), 1638, 1510, 1218, 1090, 765, 658. HRMS (ESI-TOF) calculated for C_24_H_24_IN_5_O_4_ + H^+^: 574.0946; Found: 574.0945.

*Ethyl 6-methyl-2-oxo-4-(4-(prop-2-ynyloxy)phenyl)-1,2,3,4-tetrahydropyrimidine-5-carboxylate* (**13**). In a 100 mL pressure vessel equipped with a magnetic stirrer, aldehyde **2** (160 mg, 1 mmol), urea (72 mg, 1.2 mmol), ethyl acetoacetate (0.14 mL, 1.1 mmol), and *p*-toluenesulfonic acid monohydrate (57 mg, 0.3 mmol) were added in EtOH (3 mL). The mixture was heated at 60 °C for 2 days, cooled and poured into ice/water (15 g), and the precipitate was filtered off and dried. The light yellow solid **13** (260 mg, 82% yield) was used directly for the following reaction. A sample for analysis was recrystallized from CH_2_Cl_2_-hexane (1:2 *v*/*v*), which gave a colorless solid, m.p. 159–160 °C. ^1^H-NMR (DMSO-*d*_6_): δ = 1.10 (t, *J* = 7.1 Hz, 3H, CH_3_), 2.24 (s, 3H, CH_3_), 3.52 (t, *J* = 2.2 Hz, 1H, ≡CH), 3.99 (q, *J* = 7.1 Hz, 2H, OCH_2_), 4.76 (d, *J* = 2.1 Hz, 2H, OCH_2_), 5.10 (d, *J* = 2.8 Hz, 1H, CH), 6.93 (d, *J* = 8.6 Hz, 2H, ArH), 7.16 (d, *J* = 8.6 Hz, 2H, ArH), 7.66 (br, 1H, NH), 9.14 (br, 1H, NH). ^13^C-NMR (DMSO-*d*_6_): δ = 14.6 (OCH_2_*C*H_3_), 18.2 (CH_3_), 53.8 (CH), 55.9 (OCH_2_), 59.6 (O*C*H_2_CH_3_), 78.5 (≡CH), 79.8 (C≡), 100.0 (C=), 115.2 (2 × ArCH), 127.8 (2 × ArCH), 138.3 (C_ipso_), 148.5 (=CNH), 152.6 (NC=ON), 156.9 (O-C_ipso_), 165.8 (OC=O). FT-IR/ATR ν_max_ cm^−1^: 3276, 3246, 3114, 2981, 2920, 2115 (C≡C), 1704 (C=O), 1650, 1508, 1221, 1175, 1093, 1034, 776. HRMS (ESI-TOF) calculated for C_17_H_18_N_2_O_4_ + H^+^: 315.1339; Found: 315.1344.

## 4. Conclusions

In conclusion, we have developed an efficient synthesis of 1,2,3-triazole derivatives of dihydropyrimidinones by the MCR strategy (Click/Biginelli reactions) in good yields. Consequently, this MCR strategy is a powerful synthetic tool for the construction of new corrosion inhibitors based on 1,2,3-triazoles and dihydropyrimidinones. EIS measurements showed that the inhibitive capacity for API 5 L X52 steel in 1 M HCl of the heterocycles **8**–**12** was comparable or better than that other organic inhibitors that incorporate the 1,2,3-triazole moiety. Further investigations of these heterocycles as corrosion inhibitors for steel are in progress and will be reported in due course.

## Figures and Tables

**Table 1:** One-pot three-component reaction catalyzed by Cu(OAc)_2_·H_2_O. 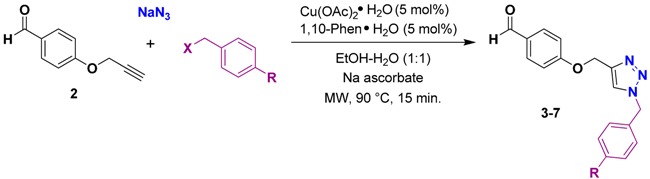

Entry ^a^	Compound	X	R	Yield ^c^ (%)
1 ^b^	**3**	Cl	H	78
2	**3**	Cl	H	82
3	**4**	Cl	F	80
4	**5**	Cl	Cl	86
5	**6**	Br	Br	75
6	**7**	Br	I	72

^a^: Reagents: **2** (1.25 mmol), NaN_3_ (1.38 mmol), and benzyl halide (1.38 mmol); ^b^: The reaction was conducted at room temperature for 12 h. ^c^: Isolated yields after purification.

**Table 2:** Biginelli reaction catalyzed by Ce(OTf)_3_. 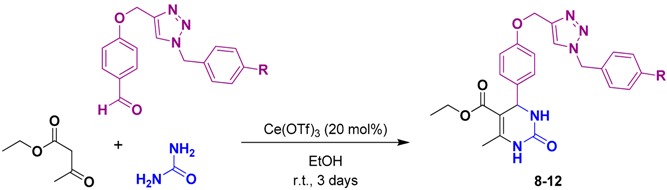

Entry ^a^	Compound	R	Yield ^b^ (%)
1	**8**	H	80
2	**9**	F	87
3	**10**	Cl	86
4	**11**	Br	85
5	**12**	I	84

^a^: Reagents: aldehydes **3**–**7** (0.34 mmol), urea (0.37 mmol), and ethyl aceto-acetate (0.37 mmol); ^b^: Isolated yields after purification.

**Table 3:** Electrochemical parameters obtained from experimental impedance data including the corrosion inhibition efficiencies (IE) at 10 ppm of the organic inhibitor.

Compound	C_dl_/µF·cm^−2^	R_ct_/Ωcm^2^	IE/%
Blank	310.0	30.0	-
**8**	38.0	618.4	95.1
**9**	83.3	564.9	94.7
**10**	34.8	733.0	95.9
**11**	61.9	573.7	94.8
**12**	49.5	678.9	95.6

## Supplementary Materials

Copies of ^1^H- and ^13^C-NMR spectra for compounds **3**–**13** (Figures S1–S11). Supplementary materials can be accessed at: http://www.mdpi.com/1420-3049/21/2/250/s1.
