# Supplementary material for: Multicomponent Synthesis and Evaluation of New 1,2,3-Triazole Derivatives of Dihydropyrimidinones as Acidic Corrosion Inhibitors for Steel
Source: Molecules. 2016 Feb 22;21(2):250. doi: 10.3390/molecules21020250 (PMC6274058; doi:10.3390/molecules21020250)
Supplement: Supplementary file 1 [file molecules-21-00250-s001.pdf]

Supplementary Materials: Multicomponent Synthesis and Evaluation of New 1,2,3-Triazole Derivatives of Dihydropyrimidinones as Acidic Corrosion Inhibitors for Steel

Rodrigo González-Olvera, Viridiana Román-Rodríguez, Guillermo E. Negrón-Silva, Araceli Espinoza-Vázquez, Francisco Javier Rodríguez-Gómez and Rosa Santillan

^{1}H NMR for compound 3.

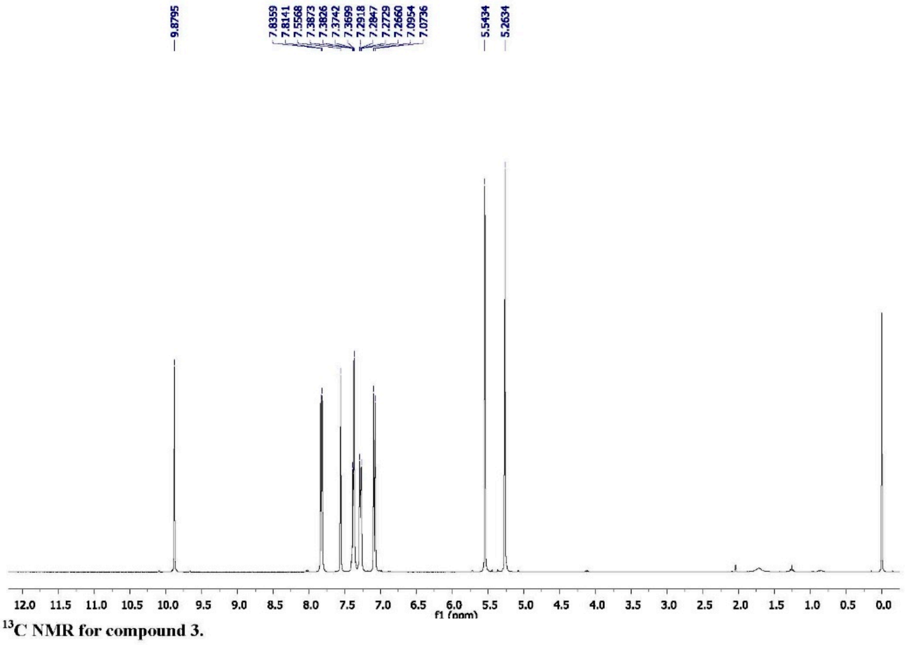

^{13}C NMR for compound 3.

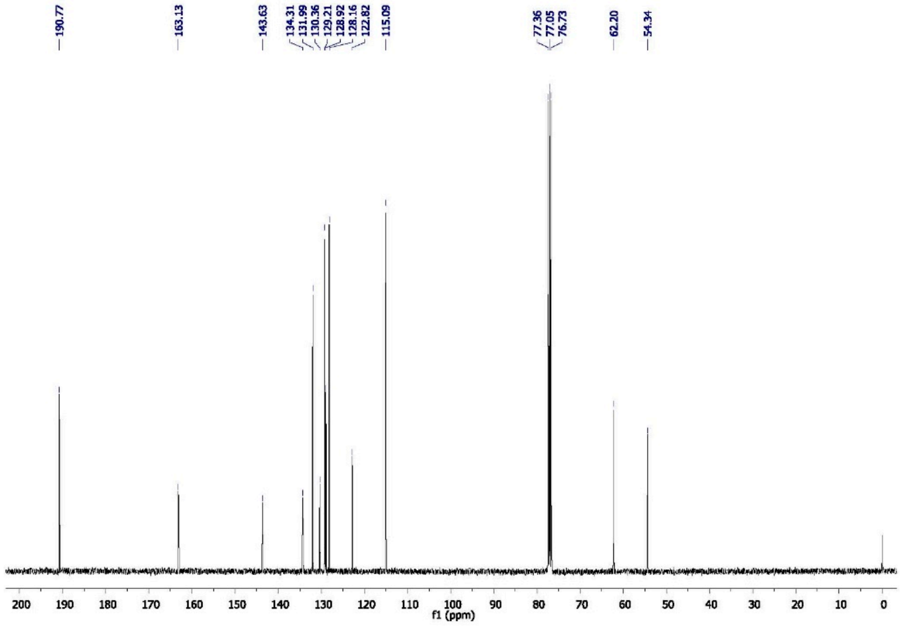

Figure S1. ^{1}H-NMR and ^{13}C-NMR for Compound **3**.

^{1}H NMR for compound 4.

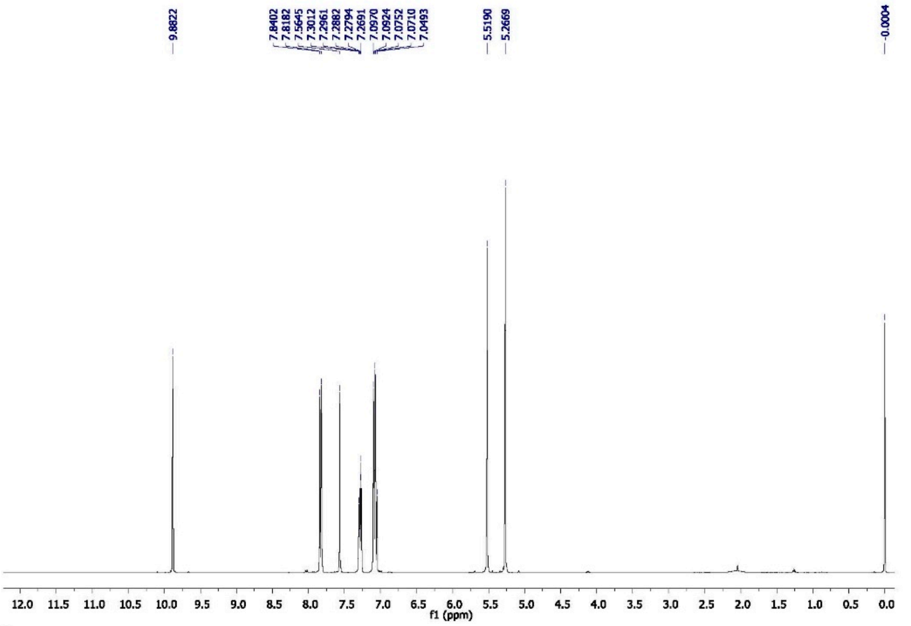

^{13}C NMR for compound 4.

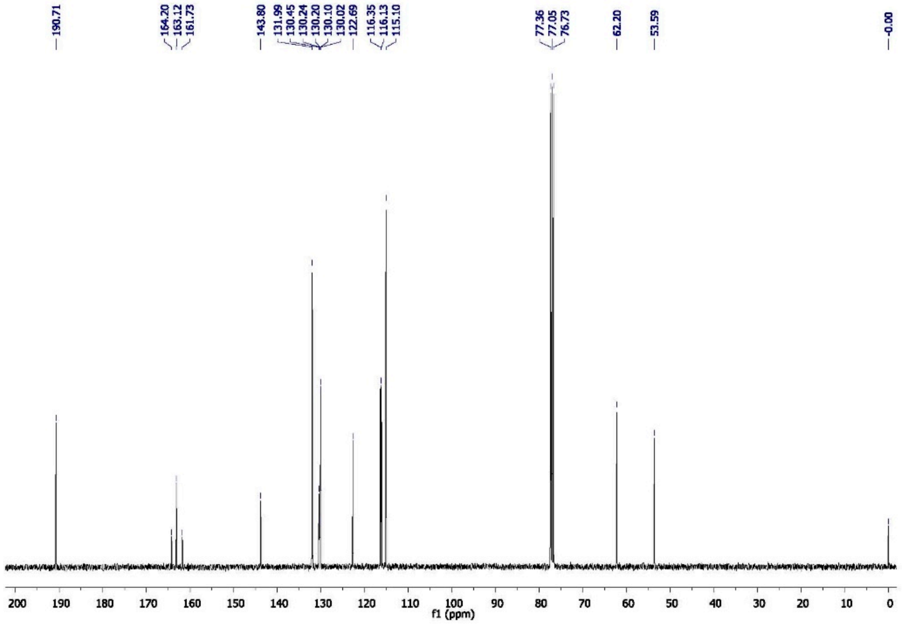

Figure S2. ^{1}H-NMR and ^{13}C-NMR for Compound **4**.

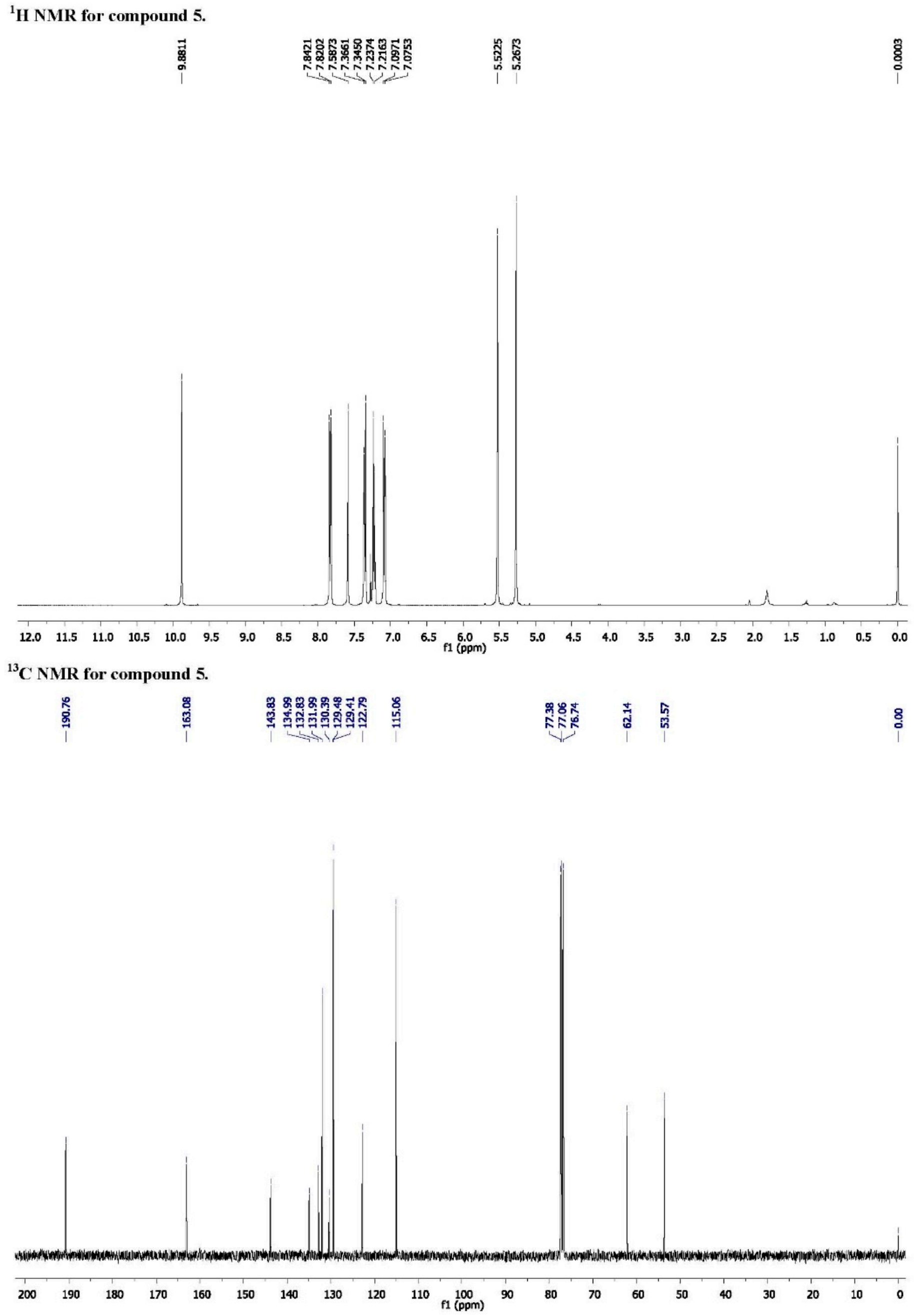

Figure S3. ^{1}H-NMR and ^{13}C-NMR for Compound **5**.

^{1}H NMR for compound 6.

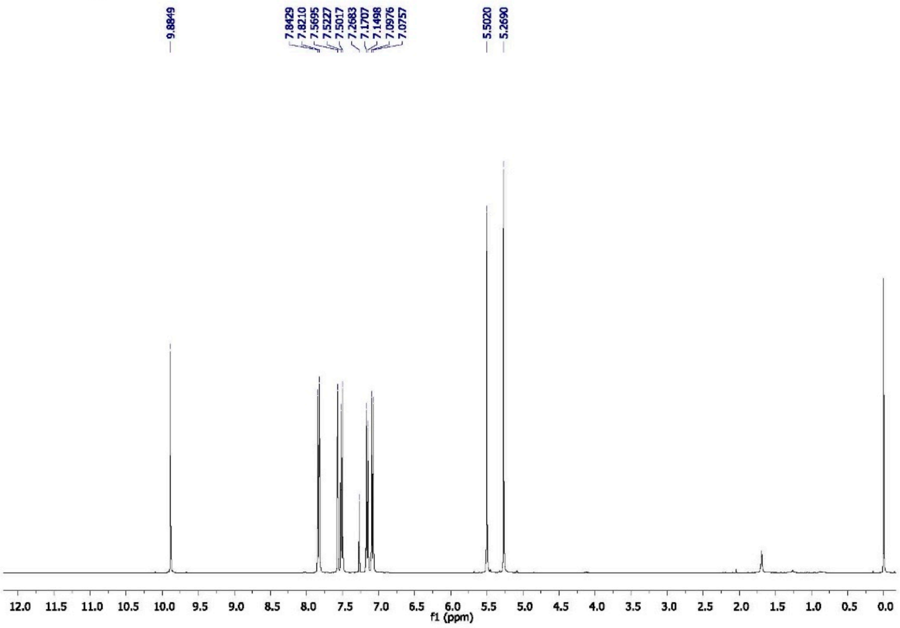

^{13}C NMR for compound 6.

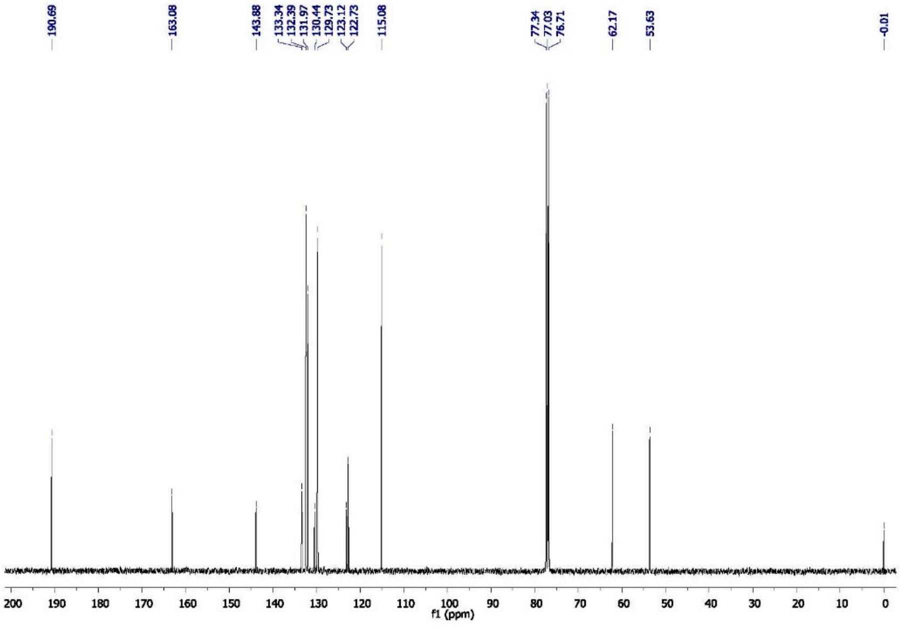

Figure S4. ^{1}H-NMR and ^{13}C-NMR for Compound **6**.

^{1}H NMR for compound 7.

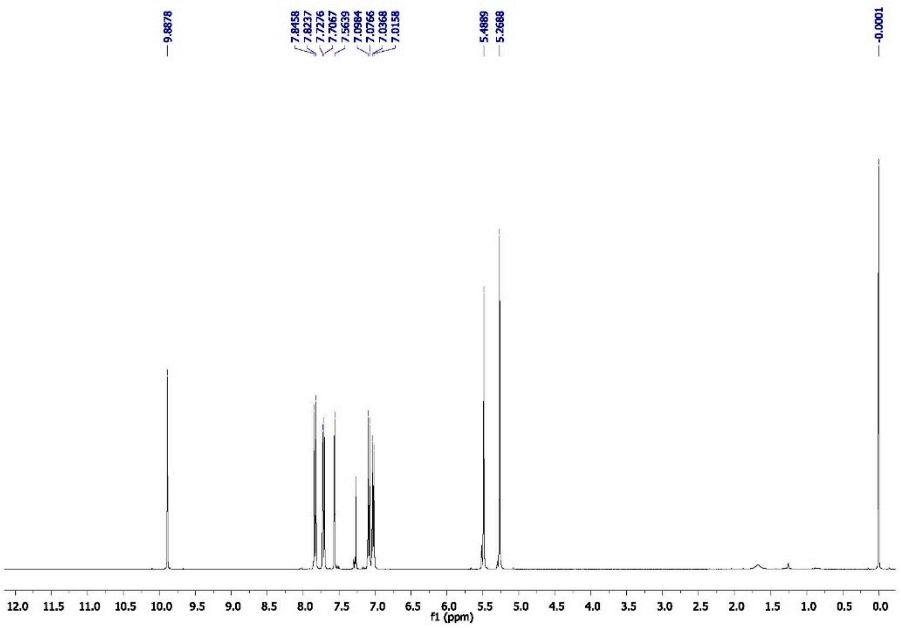

^{13}C NMR for compound 7.

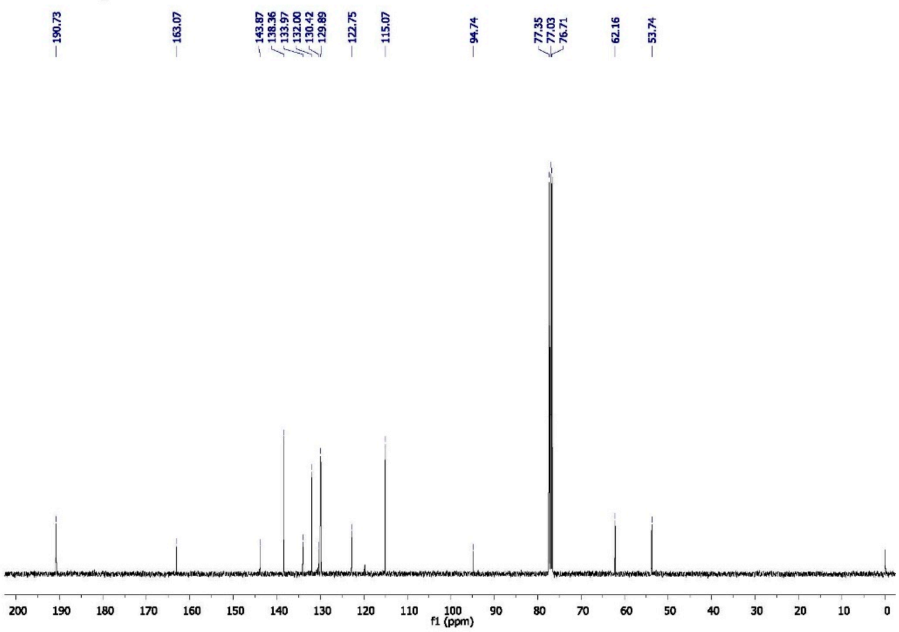

Figure S5. ^{1}H-NMR and ^{13}C-NMR for Compound **7**.

^{1}H NMR for compound 8.

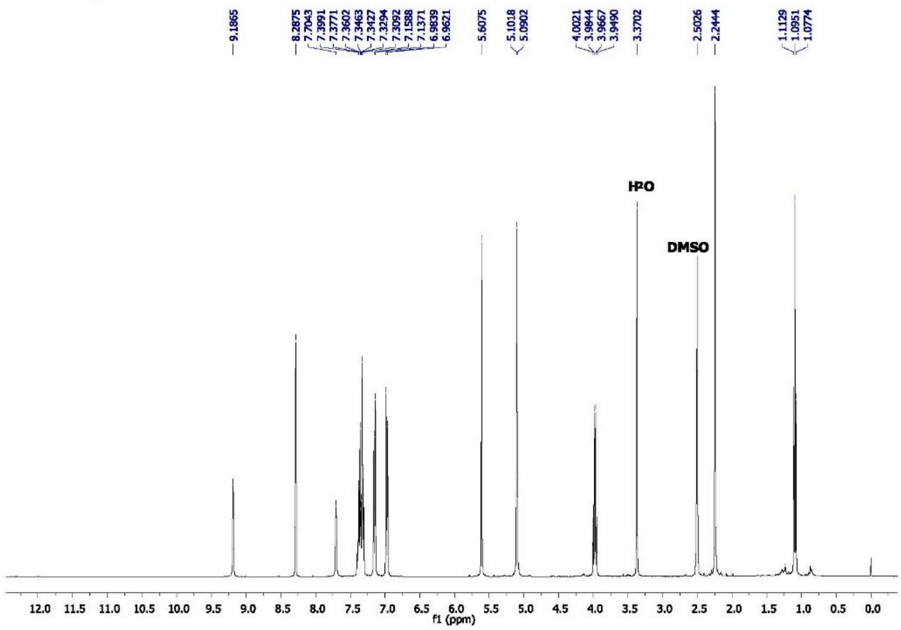

^{13}C NMR for compound 8.

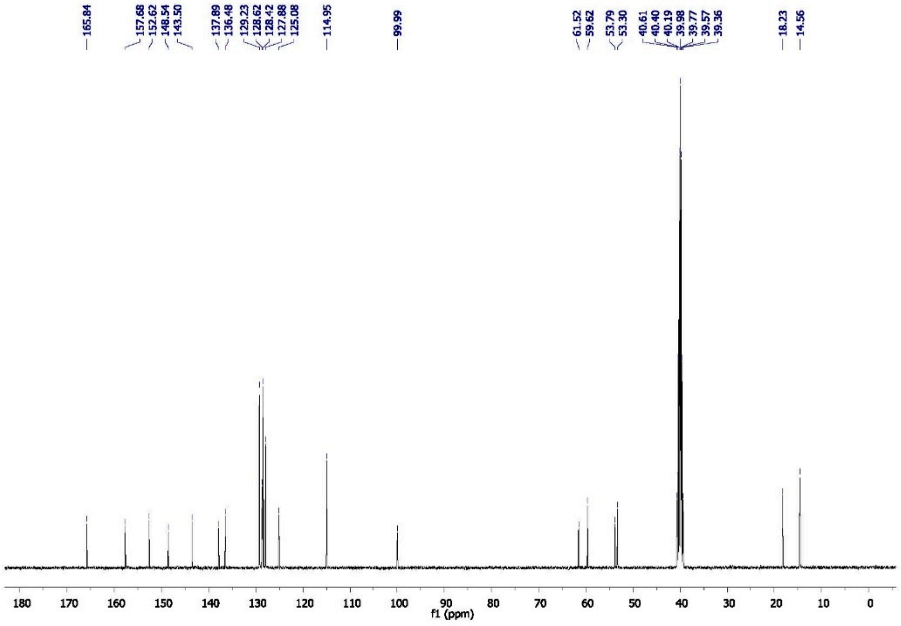

Figure S6. ^{1}H-NMR and ^{13}C-NMR for Compound **8**.

^{1}H NMR for compound 9.

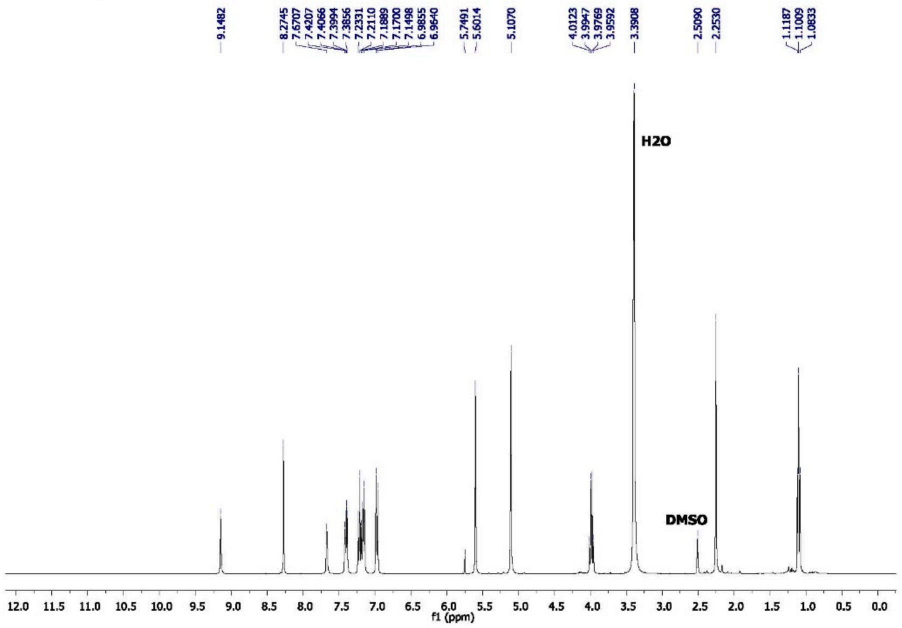

^{13}C NMR for compound 9.

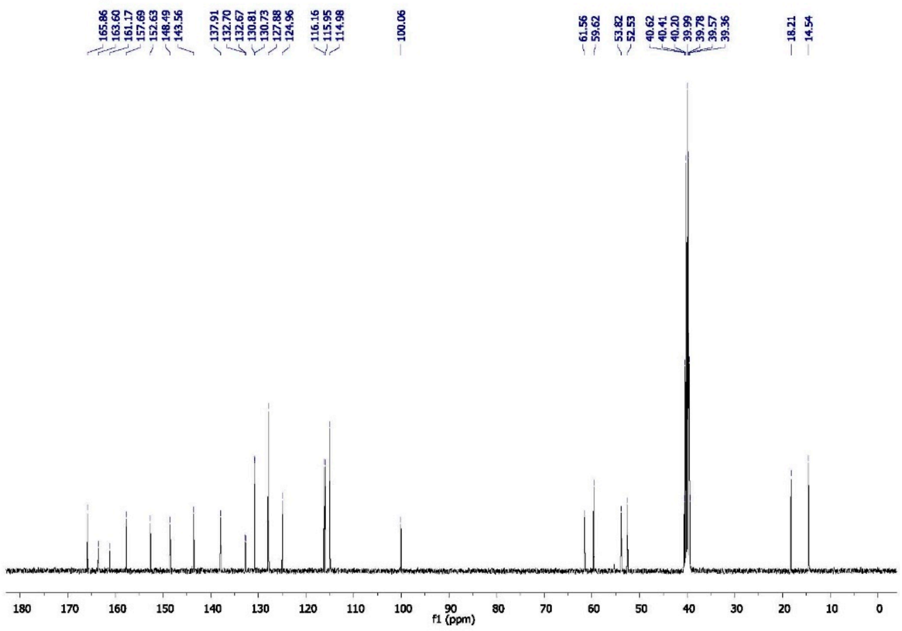

Figure S7. ^{1}H-NMR and ^{13}C-NMR for Compound **9**.

^{1}H NMR for compound 10.

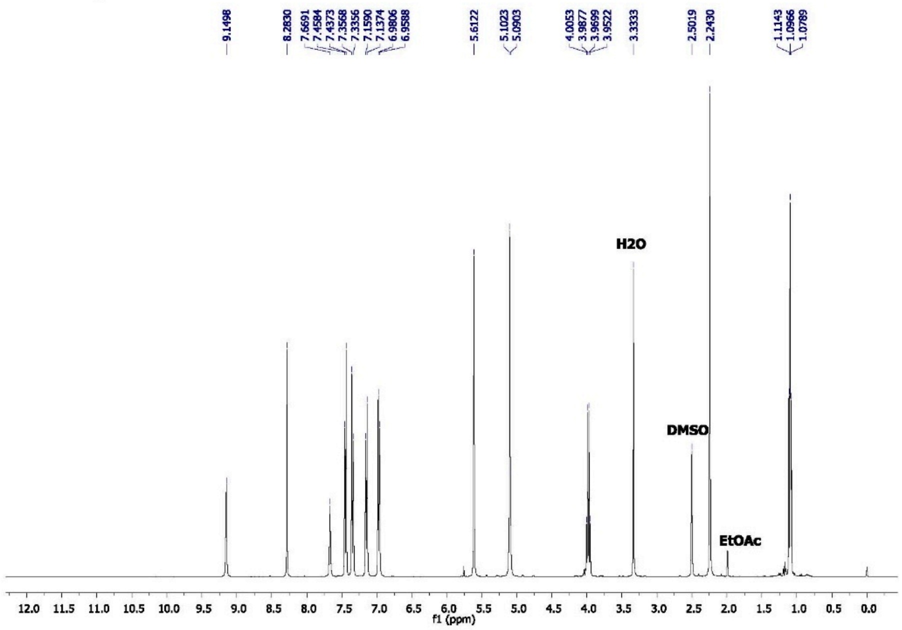

^{13}C NMR for compound 10.

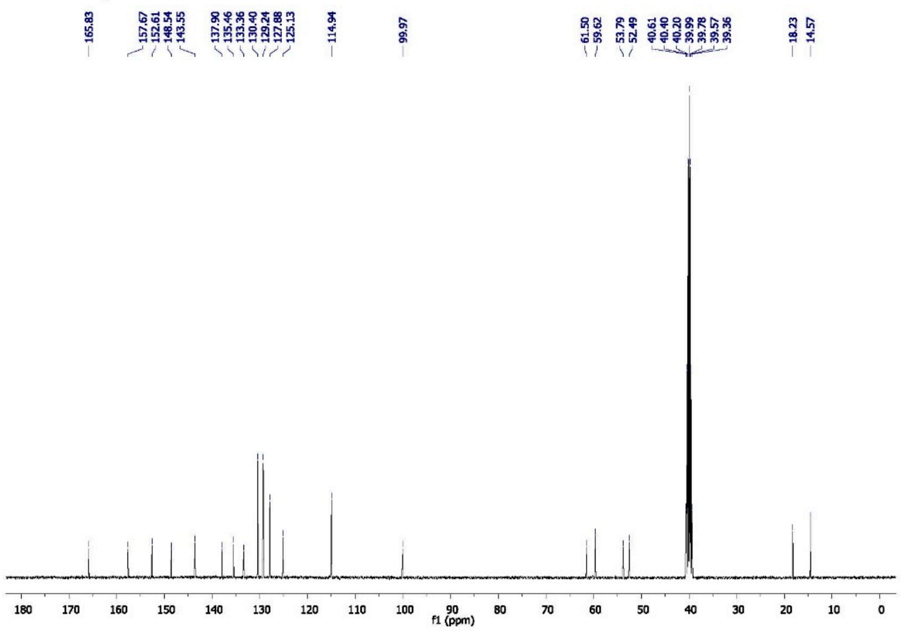

Figure S8. ^{1}H-NMR and ^{13}C-NMR for Compound **10**.

^{1}H NMR for compound 11.

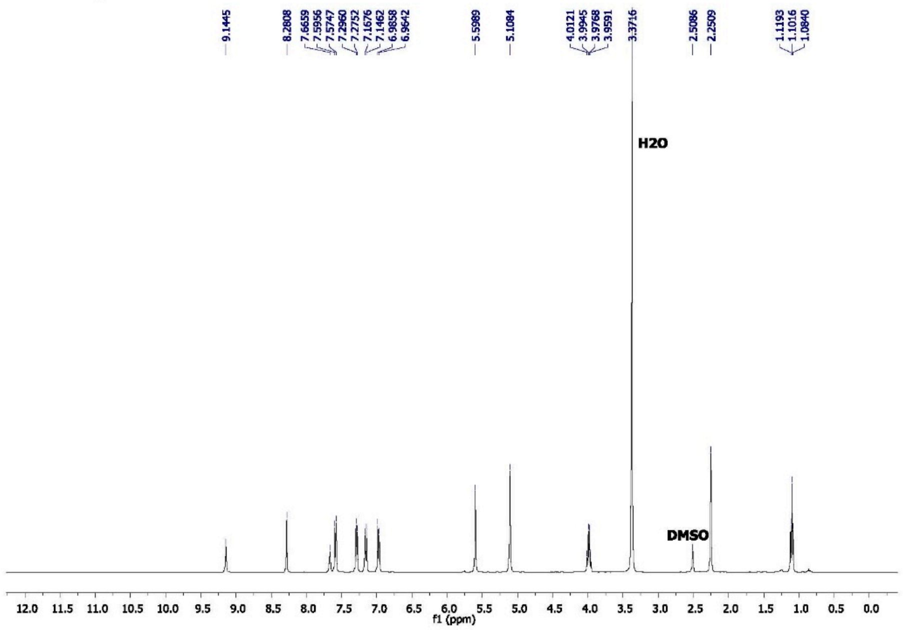

^{13}C NMR for compound 11.

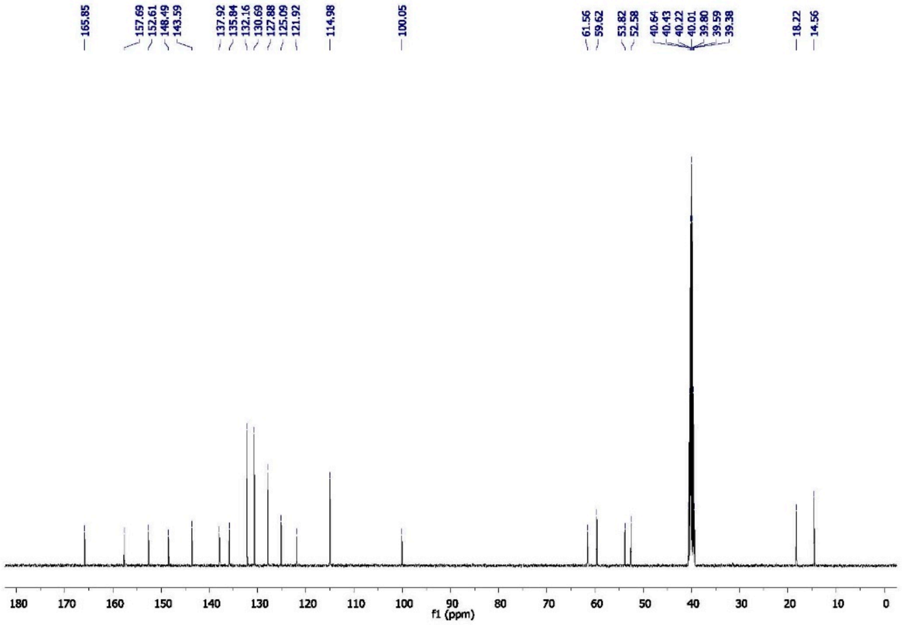

Figure S9. ^{1}H-NMR and ^{13}C-NMR for Compound **11**.

^{1}H NMR for compound 12.

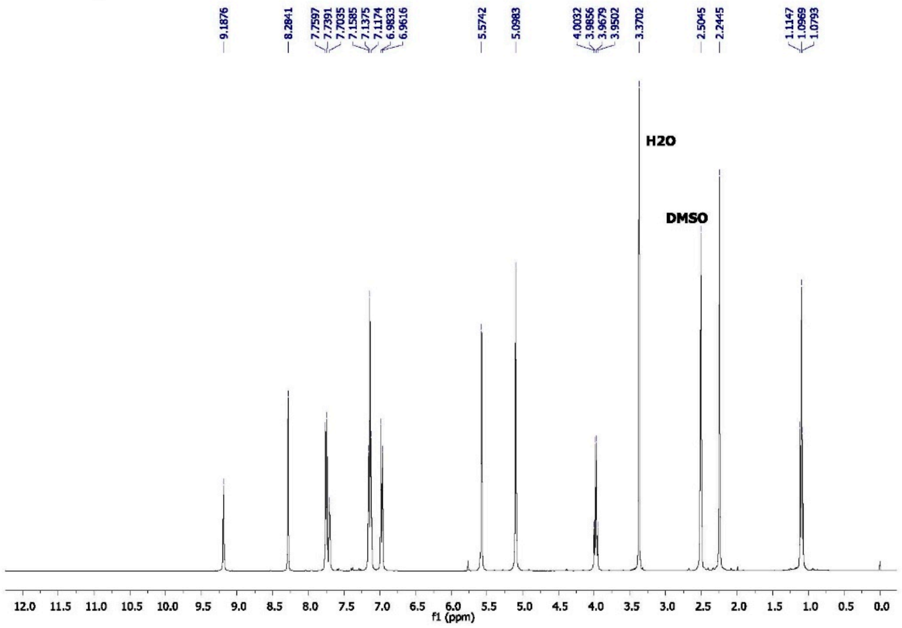

^{13}C NMR for compound 12.

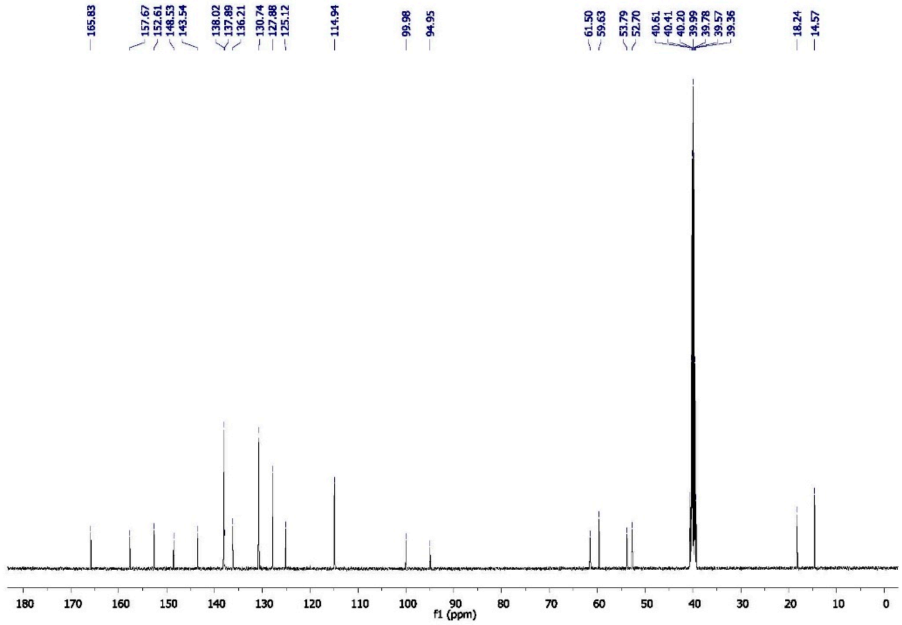

Figure S10. ^{1}H-NMR and ^{13}C-NMR for Compound **12**.

^{1}H NMR for compound 13.

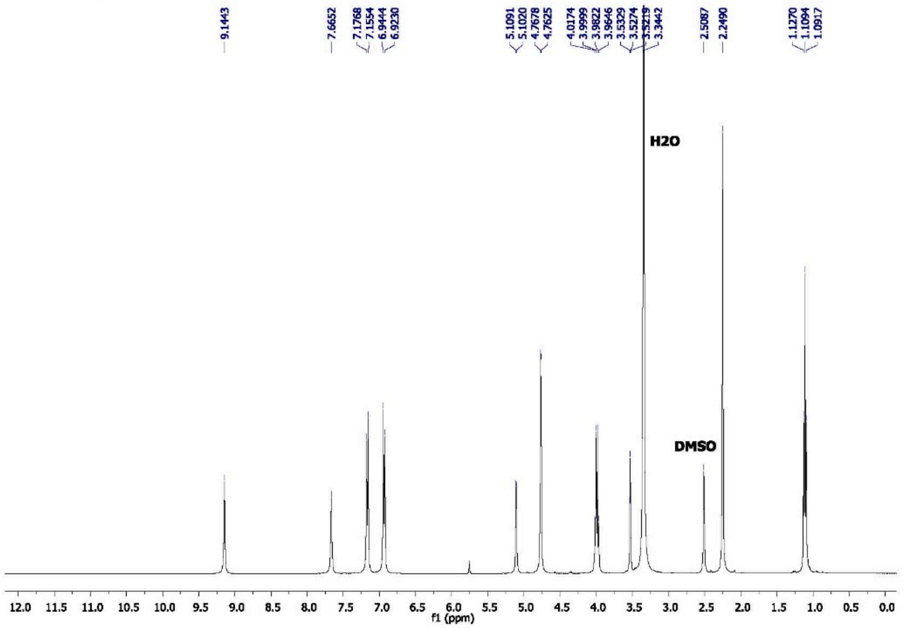

^{13}C NMR for compound 13.

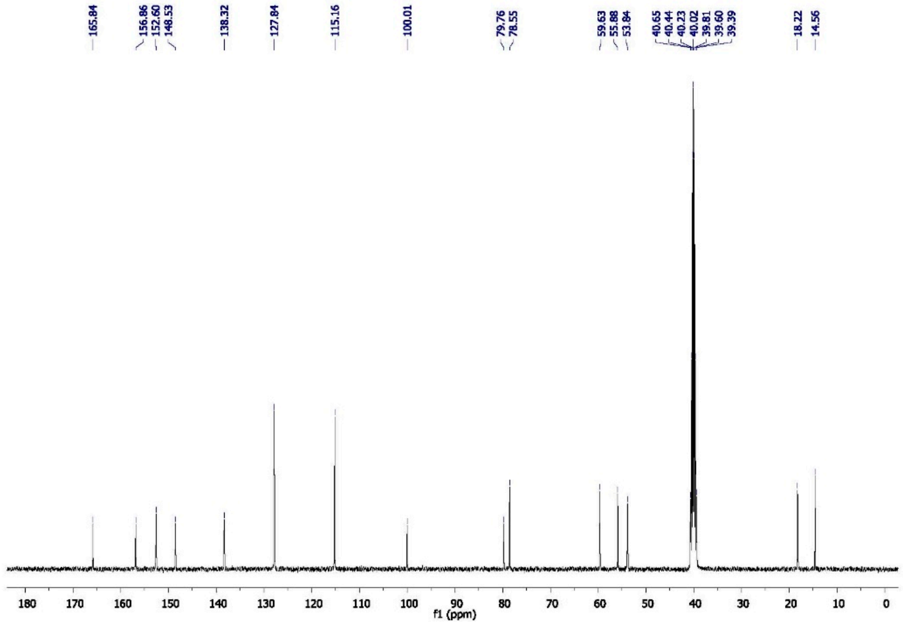

Figure S11. ^{1}H-NMR and ^{13}C-NMR for Compound **13**.
